# Predictive value of mitotic figure counts in tumor progression of non-invasive high-grade papillary urothelial carcinoma of the urinary bladder: A retrospective study from a single cancer center

**DOI:** 10.14440/bladder.2024.0021

**Published:** 2025-01-27

**Authors:** Yan Hu, Susan Karki, Weiwei Chen, Yunguang Liu, Norbert Sule, Bo Xu

**Affiliations:** 1 Department of Pathology, State University of New York, Buffalo, New York 14263, United States of America; 2 Department of Pathology, Catholic Health Care System, Buffalo, New York 14220, United States of America; 3 Department of Pathology, Roswell Park Comprehensive Cancer Center, Buffalo, New York 14263, United States of America; 4 Department of Pathology, The University of British Columbia, Vancouver V6T 1Z4, Canada; 5 Northwest Arkansas Pathology Associates, Fayetteville, Arkansas 72703, United States of America

**Keywords:** Tumor progression, Mitotic figure count, Predictive value, Bladder, Non-invasive urothelial carcinoma

## Abstract

**Background:**

Urothelial carcinoma (UC) is the most common type of bladder malignancy. Although the majority of UC present as non-invasive tumors, a subset of them progress into invasive cancer and cause significant morbidity and mortality.

**Objective:**

In this study, we examined the association between tumor mitotic activity associated and the progression of non-invasive high-grade papillary UC of the bladder.

**Methods:**

Forty-four cases of tumors that met the selection criteria were retrieved from the Department of Pathology archives, and, for each case, mitotic figures were counted in 10 high-power fields (HPF) by two independent pathologists. Tumor progression was defined as the invasion of the tumor into the subepithelial connective tissue (lamina propria) or beyond during follow-ups. In addition, tumors that later exhibited distant metastases were included in the tumor progression group.

**Results:**

Our study revealed that the average mitotic count per 10 HPF in the tumor progression group was significantly higher (*p =* 0.001) than in the progression-free group. Furthermore, tumors with more than three mitotic counts per HPF in initial biopsies posed a high risk of tumor progression within the 19.5 ± 6.1 months of follow-ups.

**Conclusion:**

The findings of our study provided valuable information for further stratification of risk factors among patients with non-invasive high-grade papillary UC of the bladder. Patients with high mitotic figure count in their initial biopsies should be monitored closely or treated earlier to prevent their tumors from progressing into invasive carcinoma.

## 1. Introduction

Globally, bladder cancer (BC) is the tenth most common cancer and the thirteenth leading cause of cancer-related deaths^1^,^2^. In the United States, BC is the fourth most common cancer in men, the eighth in women, and the fifth overall, with a man-to-woman ratio of 4:1^3^,^4^. The incidence of BC increases significantly after five decades of life. Tobacco smoking and occupational exposures (aromatic amines and polycyclic aromatic hydrocarbons) have been considered the most substantial risk factors^5^,^6^.

About 90% of bladder malignancies are urothelial carcinoma (UC). UC is associated with significant morbidity, mortality, and financial burden on the healthcare system. There is a stronger predilection for males than for females (male-to-female ratio = 6:1–8:1), and the mean age of patients is 70 years old^7^. UC can be clinically classified into two groups: superficial (non-muscle-invasive) tumors and muscle-invasive tumors. The distinction between these two groups is important, as an invasion into muscularis propria (detrusor muscle) serves as the decision point for radical cystectomy. Nevertheless, more than two-thirds of patients have non-muscle-invasive UC during initial presentation^7^. Identifying BC patients with high-risk factors for tumor progression is essential for ensuring timely and appropriate management of this lethal disease.

Histological classification of UC, including grading and staging, plays a crucial role in risk stratification of tumor recurrence, progression, and response to treatment. Non-invasive high-grade papillary UC is a urothelial neoplasm characterized by papillary configuration and moderate-to-severe architectural disorder. Cytologically, atypia of tumor cells may be severe and is characterized by large pleomorphic nuclei, prominent nucleoli, and atypical mitosis; however, there is no cellular invasion through the basement membrane. Since 2004, the World Health Organization’s (WHO) third edition of the Classification for Urinary and Male Genital Tumors deleted the ambiguous grade 2 of tumor classification and categorized UC into low or high grades, increasing the number of non-invasive high-grade UCs^7^,^8^. As heterogeneous tumor grade is reported in nearly one-third of papillary urothelial tumors, the WHO 2016 and 2024 editions recommended that if the high-grade area is <5% of the total tumor volume, the tumor should be reported as “low-grade with <5% high-grade component.” Conversely, if the high-grade component is >5%, then the tumor should be reported as high-grade^9^-^11^.

Tumor cell proliferation is a key indicator of tumor progression. The Ki-67/MIB-1 protein is a cellular marker for cell proliferation, detectable within the cell nucleus using the monoclonal antibody MIB-1. It is considered a predictive factor for tumor development. Ki-67 is present during all active phases of the cell cycle (G1, S, G2, and mitosis) but is absent from resting cells (G0)^12^. Proliferative activity, evaluated in terms of mitotic figure count, Ki-67, or MIB immunohistochemistry (IHC), is a significant indicator of the biological behavior of UC^13^. These markers have been shown to serve as independent prognostic markers of patient outcomes in several malignancies^14^-^16^, correlating with the recurrence, progression, and survival outcomes of UC. The Ki-67 level increases from papilloma to papillary urothelial neoplasm of low malignant potential, as well as in low-grade and high-grade non-invasive tumors. High-level tumor proliferation is correlated with tumor size, recurrence, progression, and survival outcomes^17^. In cases of muscle-invasive urinary BC following radical cystectomy, the Ki-67 index has proven to be a useful biomarker for predicting oncological outcomes. However, absolute cut-off values for predicting tumor progression in non-invasive high-grade papillary UC have not yet been established. In this study, we investigated if mitotic activity is associated with tumor progression in non-invasive high-grade papillary UC of the urinary bladder.

## 2. Materials and methods

### 2.1. Case selection

This study was a single-center, retrospective analysis approved by the Institutional Review Board. Forty-four cases of non-invasive high-grade papillary UC identified in initial biopsies and follow-ups at our hospital were retrieved. The cases were sourced from the Roswell Park Comprehensive Cancer Center pathology archive between the years 2010 and 2020. Slides of the initial biopsies and follow-ups were reviewed, and cases without follow-up data were excluded from the analysis.

### 2.2. Histological grading

Slides were stained with hematoxylin and eosin (H&E) and subsequently reviewed and graded based on the most poorly differentiated areas. Tumor grading was performed according to the WHO 2016 and 2022 classification for genitourinary tumors. Tumor progression is defined as invasion into the lamina propria or beyond or the presence of distant metastasis during follow-ups.

### 2.3. Mitotic count

Areas with the highest mitotic activity (hot spots) were selected for mitotic counts at 400× magnification for each case. Mitotic figure counts were examined in 10 high-power fields (HPF) on H&E sections to ensure consistent counting in most representative spots.

### 2.4. Statistical analyses

Continuous variables were reported as mean ± standard error (SE). Results were analyzed statistically using a two-tailed *t*-test and Chi-square test. For all tests, a *p* < 0.05 was considered statistically significant. Statistical analysis was carried out using “stats” package as part of R for Windows, version 4.2.0 (R Foundation for Statistical Computing, Vienna, Austria. https://www.R-project.org/).

## 3. Results

**Figure 1:**
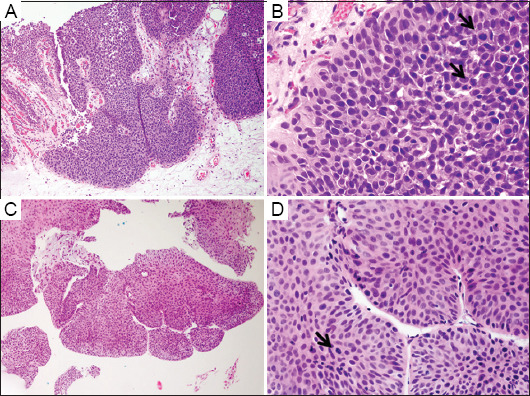
Non-invasive high-grade papillary urothelial carcinoma (UC) on initial biopsy in groups with and without tumor progression. A (×100), B (×400), Non-invasive UC with progression. C (×100), D (×400), Non-invasive UC without progression. Arrows indicate mitotic figures.

The age of the 44 cases (35 males and nine females) ranged from 42 to 92 years old, with a mean age of 68 (Table 1). Histologically, all cases were graded as high-grade papillary UC on initial biopsies against the 2016 and 2022 WHO/International Society of Urological Pathology criteria. Tumor cells lining the papillary fronds showed both architectural and cytological abnormalities. Altered polarity and pleomorphic nuclei were readily observed (Figure 1). Mitotic figures were frequent, with atypical mitosis noted in some cases. The mitotic counts in all initial biopsy specimens ranged from 0 to 13 per 10 HPF, with an average count of 2.6 ± 0.5 per HPF. Follow-up of patients was done for a period ranging from 6 to 70 months, with a mean follow-up time of 37 months. During this follow-up, 10 cases (22.7%) experienced tumor progression with an average timeframe of 10.4 ±3.5 months. Progression included invasions into the lamina propria (1/10), muscularis propria (3/10), perivesical tissue and prostate (2/10), as well as metastases to the omentum (1/10), lymph node (1/10), lung (1/10), and retroperitoneum (1/10). In contrast, the remaining 34 cases (77.3%) showed no evidence of tumor progression during the follow-up, averaging 31.6 ± 3.61 months. The clinical and histological features of all 44 cases are summarized in Table 1.

**Table 1:** Clinical and histological features of the 44 patients included in this study

Clinical and histological features	Values/Description
Total number of cases	44
Mean age (range) (in years)	68 (42–92)
Number of males	35
Number of females	9
Male/female ratio	3.9
Initial diagnosis	Non-invasive high-grade papillary urothelial carcinoma
Mean mitotic counts (range)	2.6 (0–13)
Mean follow-up time (range) (in months)	37 (6–70)
Follow-up method	Biopsy, TUR, cystectomy
Number of non-progressive cases (%)	34 (77.3)
Number of progressive cases (%)	10 (22.7)

TUR: Transurethral resection.

**Figure 2:**
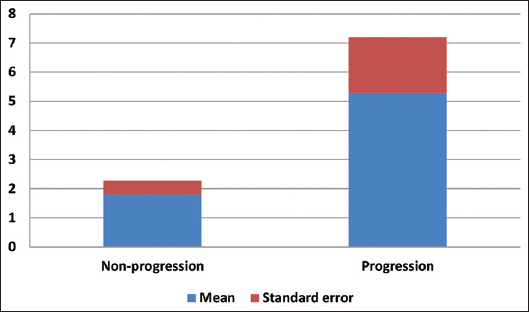
Average mitotic counts in groups with and without tumor progression.

To investigate the relationship between mitosis and tumor progression in initial non-invasive high-grade UC, we divided the cases into a progressive group (10 cases) and a non-progressive (34 cases) group. Grouping was done based on the presence of invasion of lamina propria or beyond during follow-up procedures. The average mitotic count per 10 HPF in the group without tumor progression and the group with tumor progression was 1.8 ± 0.48 and 5.3 ± 1.09, respectively. The mitotic count of tumor progression group was significantly higher than that of the group without tumor progression (p = 0.001) (Table 2 and Figure 2). In the group with tumor progression, more brisk and atypical mitotic activity was observed (Figure 1). High mitotic counts in the initial biopsies were associated with a worse prognosis. However, no significant differences related to age or gender were found to be associated with tumor progression (Table 2).

**Table 2:** Comparison of characteristic features between non-progressive and progressive groups

Characteristic features	Non-progressive group	Progressive group	*p*-value
Number of cases (%)	34 (77.3)	10 (22.7)	
Male/Female ratio	3.9	4	
Follow-up time (mean±SE) (in months)	31.6±3.61	10.4±3.48	
Age (mean±SE) (in years)	68.3±1.89	66.3±4.24	0.3
Mitotic figure/10 HPF	1.8±0.48	5.3±1.09	0.001*

Note: *Indicates statistical significance at *p<*0.05. HPF: High-power fields.

**Figure 3:**
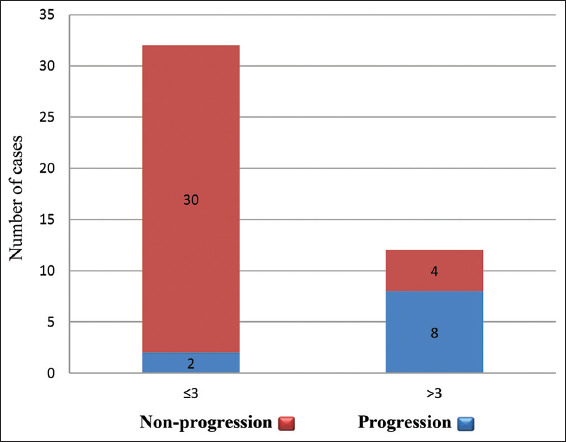
High mitotic count is associated with the elevated risk of tumor progression. Notes: Red bar: Non-progression; Blue bar: Progression.

To further evaluate the correlation between mitotic figure counts and tumor progression, the cases were divided into two groups based on mitotic counts: group one (>three mitotic figure counts per 10 HPF) and group two (≤three mitotic figure counts per 10 HPF). Eight out of 12 cases in group one showed tumor progression with an average follow-up of 19.5 ± 6.1 months. In contrast, only two out of 32 cases in group two exhibited tumor progression with an average follow-up time of 29.2 ± 3.7 months. The relative risk for tumor progression for biopsy specimens containing more than three mitotic figure counts was 10.7, with a 95% confidence interval^2^,^6^ (Figure 3).

## 4. Discussion

UC falls into two categories: non-muscle invasive (superficial) and muscle-invasive tumors. Most of the superficial UC are non-invasive papillary tumors at initial presentation, with approximately 70% of these superficial papillary tumors recurring over a prolonged clinical course, leading to significant morbidity^15^. Ki-67 is an established cell proliferation marker that is active during the G1, S, G2, and M phases of the cell cycle. Amin *et al*. found that high-grade papillary UC had increased expressions of p53 and MIB-1 compared to their low-grade counterparts^15^. Conventional prognostic factors, such as tumor grades and stages, have limited predictive value with non-invasive high-grade UC. Therefore, a reliable approach to predicting tumor aggressiveness is needed for stratifying risk factors associated with tumor progression.

In this study, we assessed the association between mitotic figure counts and tumor progression in non-invasive high-grade papillary UC of the bladder. Our findings demonstrated a significant positive correlation between mitotic counts and tumor progression. Specifically, our data revealed that increased mitotic activity (>three mitotic figure counts per 10 HPF) in initial biopsy specimens was associated with a higher risk of progression into invasive tumors and distant metastases.

Mitotic count estimates have been widely used as a simple method to measure tumor cell proliferation in histological sections, particularly when compared to the Ki-67 proliferating index obtained through IHC staining, which marks all phases of the cell cycle except G0^17^. Goyal *et al*. reported that a Ki-67 index of ≥59% and mitotic counts of ≥36.50/10 HPF were 100% specific for invasive BC. This finding has practical utility in tumor staging and management, especially in cases where morphological evidence of muscle invasion is equivocal^18^. Another study showed that areas of high-grade UC displayed strong and diffuse Ki-67 reactivity in 20–0% of the tumor, while low-grade UC areas had negative or focal reactivity for Ki67 in 10–30% of the tumor^19^. In our study, we investigated the predictive value of mitotic counts in non-invasive high-grade UC and found that a cut-off value of mitotic counts greater than three per 10 HPF in initial biopsy specimens strongly correlated with tumor progression and distant metastasis.

The results of our study added more to the risk stratification for progression in non-invasive high-grade papillary UC. The introduction of artificial intelligent-assisted counting of mitotic figures in hot spots will enhance the objectivity and reliability of diagnosis and patient management compared to conventional grading and staging alone^20^.

This study was subject to several limitations. This study had a relatively small number of cases and a short follow-up time with some patients. A multicenter collaboration involving a larger cohort and extended follow-ups is warranted to fully unravel the roles of mitosis and other biomarkers in the biological progression of UC.

## 5. Conclusion

This study reveals the implication of mitotic counts in tumor progression of non-invasive UC. Patients with high mitotic figure count in their initial biopsies should be monitored closely or treated earlier to prevent tumors from progressing into invasive carcinoma.

## Data Availability

All data are presented in this article.
